# Risky motorcycle riding behaviour among young riders in Manipal, India

**DOI:** 10.1186/s12889-021-11899-y

**Published:** 2021-10-28

**Authors:** Kumar Sumit, Veerle Ross, Kris Brijs, Geert Wets, Robert A. C. Ruiter

**Affiliations:** 1 Maastricht University, Department of Work & Social Psychology, Faculty of Psychology and Neuroscience, Maastricht University, P.O. Box 616, 6200 MD Maastricht, The Netherlands; 2 UHasselt, School of Transportation Sciences, Transportation Research Institute (IMOB), Agoralaan, 3590 Diepenbeek, Belgium; 3 Prasanna School of Public Health, Manipal Academy of Higher Education, Manipal, India

**Keywords:** Motorcycle rider behaviour questionnaire, Manipal, Road crashes, Young riders

## Abstract

**Background:**

Motorcycles are one of the most commonly used transportation modes in low and middle-income countries. In India, motorized two-wheelers comprise 70% of the total vehicle population, and motorcycle users are considered the most vulnerable road users. It is essential to understand the risky riding behaviour and associated factors among the motorcyclists to develop evidence-based traffic safety programs targeting motorcycle riders. The purpose of the current study was two-fold. First, it aimed to determine the appropriate structure of a modified version of the MRBQ among young riders in Manipal, India. Second, it assessed to what extent MRBQ factors were associated with self-reported crash involvement and violations.

**Methods:**

The motorcycle rider behaviour questionnaire (MRBQ) is a 43-item scale that assesses five aspects of risky motorcycle rider behaviour, i.e., violations, control errors, traffic errors, stunts, and protective equipment. The MRBQ, along with measures of socio-demographic variables and the number of motorcycle crashes, was filled out by 300 young motorcycle riders who were in the age group of 18–25 years and had been riding for at least the past three years (93% males, 92.3% students).

**Results:**

Five factors emerged out of the MRBQ after an exploratory factor analysis: traffic errors, control errors, stunts, protective equipment, and violations. Cronbach’s alpha for these factors ranged from .66 to .82. Reports of performing stunts and committing violations were positively associated with self-reported near-crash experiences over the past three months. Riders reporting stunts, violations and using a motorcycle of 125-200 cc reported having received more fines in the last three months. These findings were confirmed in both univariate and multivariate binary logistic regression models.

**Conclusion:**

The study assessed the factor structure of a modified version MRBQ and the extracted factors associations with self-reported crash involvement. The factor structure revealed in the current study is consistent with MRBQ factor structures found in other countries. However, the support for a relationship between MRBQ factors and self-reported crashes was less significant. The findings suggest that if replicated by future studies, local policymakers are advised to focus on the five MRBQ factors while planning future interventions to achieve a reduction in the number of road crashes among motorcyclists.

**Supplementary Information:**

The online version contains supplementary material available at 10.1186/s12889-021-11899-y.

## Background

Every year, more than 1.2 million people lose their life due to a road traffic crash, making it one of the leading causes of death worldwide [1]. Around 90% of the crashes occur in low and middle-income countries (LMIC) even though their contribution to the number of vehicles in the world is 54% [1]. In India, road crashes are the sixth leading cause of death, causing immense socio-economic losses among the young aged population of the country [1, 2]. The economic growth in India has contributed to a sharp increase in the motorization of transportation. This surge in motorization, coupled with the expansion of the road network, has brought with it the challenge of addressing adverse factors such as the increase in road crashes.

The national crime records bureau of India indicated that around 150,785 people were killed, and 494,624 were injured due to road traffic injuries in 2016 [3]. There has been a fourfold increase in the number of road traffic crashes in India during the last four decades accompanied by 9.8 times increase in the fatalities associated with road crashes. Road traffic injuries place a massive burden on the health care sector in terms of hospitalization and rehabilitation [2]. In a report published by the Government of India, 21.1 and 23.2% of fatal crash victims were in the age group of 18–25 years in 2016 and 2017, respectively [3].

Motorized two-wheelers comprise 70% of the total vehicle population in India. In India, as per the Motor Vehicles act of 1988, applications for the provisional driving license can be made from the age of 16 years. An applicant has to attain the age of 18 to start the licensing procedure for a geared motorcycle with an engine capacity of 100 cc or above. There is no doubt that riding a motorcycle is the most common mode of transportation in India and motorcyclists are vulnerable to road traffic injuries (RTIs), even more so because in case of a collision, their bodies are exposed directly to an obstacle or another vehicle [2]. This vulnerability is reflected in the crash statistics from India, where motorcyclists account for a substantial share of crashes with 33.8% in 2016 and 33.9% in 2017, respectively [3].

Young novice riders form a target group of interest due to two factors, i.e., experience and age [4]. First, crash risk is higher for young drivers due to lack of experience, for instance, in comprehending, assessing and responding to hazards [5]. Similar issues with experience could be at play for inexperienced motorcycle riders. Second, related to age, risky driving amongst young drivers has been theoretically explained by neurocognitive evidence that suggests an imbalance between the development of the social-affective brain and the cognitive control system during the transition period from child to adult [6]. The brain’s socio-emotional reward system shows early adolescent remodelling while the cognitive control system (e.g., inhibitory control, working memory, mental flexibility, and planning) matures more gradually, well into people’s 20s. This maturational gap between both brain systems makes it difficult for youngsters to self-regulate impulsive responses, which is even more pronounced in males than in females. One explanation for this sex difference is that male road users, compared with female road users, prioritize the benefits of risk taking over the costs associated with it [6, 7]. Again, the same could be applied to young motorcycle riders as well. Young adult riders were indeed found to be prone to risk taking in response to highly social-affective situations such as the presence of a peer passenger or riding highly powered motorcycles [8]. Furthermore, in a case-control study conducted by Mullin et al., among motorcyclists, it was reported that there was a strong and consistent negative relationship between riders’ age and their risk of moderate to fatal injuries [9].

Globally, road crashes have become the 8th leading cause of death worldwide for all age groups and the 1st leading cause of death among the young aged population [1]. Riders in the age range of 18–25 years contribute to 41.4% of India’s total road crash victims [3]. Several studies conducted in India [2, 9 10] have indicated the vulnerability of young riders for road crashes. In Udupi district, with the university town Manipal at its centre and where the current study took place, there were 787 crashes with motorcyclists in the first half of 2015, among which 41 school and college-going students [10]. Nearly 10% of all victims in traffic were in the age range of 18–25 years, and 33% of them were motorcycle users [10]. It should be noted that many of the road crashes cases in India are underreported due to less awareness about the reporting procedure or informal settlement of road crashes between the parties involved [11].

Individual rider characteristics such as young age, male gender, low economic and social status, and risky behaviours such as speed violations, not obeying traffic laws, competing with other fellow riders are the key factors causing motorcycle crashes among young riders [12–15]. Risky rider behaviours may have different psychological motives because those behaviours might be intentional or nonintentional. Behaviours like not allowing enough time to stop at traffic lights, making inappropriate headway may result from inexperience and are hence considered as nonintentional behaviours [16]. Behaviours such as speeding, riding under the influence of alcohol, not wearing protective clothing are mostly conscious related decisions and thus might be considered intentional, although instances can be identified in which for example speeding is not intentional, for example because riders are not aware of the speed limit for a specific road section or do not regularly attend to the speedometer [14, 17]. Also, a distinction is made in the literature between violations and errors, where the former originate from social and motivational factors such as operational procedures, codes of practices rules, and norms whereas the latter are the results from failures in information processing by the individual resulting in slips, lapses, and mistakes [18, 19].

To bring forward interventions aimed at reducing road crashes, the behaviours that are associated with crash risks among motorcyclists need to be thoroughly understood. To this end, Elliot et al. [15] developed the motorcycle rider behaviour questionnaire (MRBQ) based on the driver behaviour questionnaire (DBQ) that has been widely used, for example to assess driver behaviour in relation to crash involvement among truck drivers [17] and car drivers [20] in different countries. Studies show that participants who have a higher score on the DBQ are more likely to be involved in crashes in the past as well as in the future [21, 22].

The need for a tool that enhances the understanding of the involvement of human factors in motorcycle crashes led to the development of the MRBQ. Relevant items for motorcyclists were selected from the DBQ and also new items explicitly related to motorcycling were added to develop the MRBQ [15]. The MRBQ was developed along similar lines with DBQ to measure errors and violations in motorcycle riding behaviour. It also included a domain on the use of motorcycle protective equipment.

Specifically, the MRBQ contains 43 items using six-point Likert scales (1 = never, 2 = hardly ever, 3 = occasionally, 4 = quite often, 5 = frequently, and 6 = nearly all the time) that was developed to assess five aspects of risky motorcycle rider behaviour, i.e., violations (e.g., fail to notice or anticipate that another vehicle might pull out in front of you and have difficulty stopping), traffic errors (e.g., going quite wide from the corner of the road when negotiating a corner), control errors (e.g., did not notice or anticipate another vehicle coming in front of you and had difficulty to stop), stunts (e.g., intentionally do a wheel spin), and wearing protective equipment (e.g., use riding boots) [15].

The MRBQ study conducted in Turkey by Özkan et al. [23] *n* = 451; respondent’s mean age = 33.94 years; males = 100%) among commuting motorcyclists reported five-factor MRBQ structure. A similar factor structure was also reported in a study conducted by Stephens et al. [14] in Queensland, Australia (*n* = 470, respondent’s mean age = 35.4 years; males = 89%). Contrary to that a four-factor solution combining control error and traffic error was reported in another study conducted by Sakashita et al. in Victoria, Australia (*n* = 1302; respondent’s mean age = 36.0 years; males = 79.2%) among novice riders [24]. The study by Sunday et al. in Nigeria among commuting motorcyclists also revealed a four-factor structure (*n* = 500; respondent’s mean age = 27.0 years; males = 100%) [25]. The application of MRBQ lies in identifying behaviours that increases the likelihood of motorcycle crashes. For example, speed violations and control/traffic errors are the significant behavioural factors affecting motorcyclists’ crash risk [25]. Nevertheless, performing stunts was the unique MRBQ factor that correlated with crash involvement among Australian motorcyclists in Queensland [14]. Similarly, this factor was the primary determinant of active crashes (i.e., hitting another road user or an obstacle) and traffic offences (related to parking, overtaking, speeding or other traffic violations) for Turkish riders [23]. To date, most of the MRBQ studies have been conducted in high-income countries like Australia and the United Kingdom [14, 15, 24] or in countries where motorcycling is used for adventure riding or pleasure-seeking as in Turkey [23]. It is important to investigate risky riding behaviour in LMICs like India where motorcycles are the primary mode of commuting [3].

To the best of our knowledge, the MRBQ has not been tested in India yet. Therefore, the current study aimed to examine the factor structure of a modified version MRBQ among young motorcycle riders for use in India. In addition, we assessed whether the extracted MRBQ factors were associated with self-reported crash involvement, violations and the number of fines paid to examine the MRBQ’s potential in predicting risky riding behaviour among the young riders.

## Method

### Participants and procedure

Study participants were young motorcyclists in Manipal, a locality of Udupi city, which is situated at the southwest coast of India bordering the Arabian Sea in the province Karnataka. Manipal is an international university town that is home to 30,000 students from all corners of India and 60 countries across the world. It is one of the fastest-growing cities of India. The main eligibility criteria for study participation were that participants (1) should have a motorcycle of 100 cc or above, (2) either hold a motorcycle learners’ license or permanent license, and (3) have been riding regularly for the past three years.

The data collection period ranged from March to September 2018. A convenience sampling technique was used in this study to recruit 300 young motorcyclists in the age range of 18–25 years from the regional transport office and various colleges in the city. Convenience sampling was chosen because participant recruitment was being subjected to the opening hours of the colleges and the road transport office. The questionnaire was given to eligible participants in hard copy paper format and was filled out by the participants themselves after taking written informed consent from the participants. To maintain confidentiality and anonymity no names were recorded. The study was approved by the institutional ethical committee of Kasturba Medical College at Manipal Academy of Higher Education, Manipal, India (Reference number-09/2018). Since the study involved human participants, the data collection was performed in accordance with the principles of the Helsinki declaration.

### Motorcycle rider behaviour questionnaire (MRBQ)

The modified MRBQ for use in India was piloted among ten motorcyclists in the age range of 18–25 years to check the suitability of the research instrument and to assess content validity. To this end, the original MRBQ questionnaire developed by Elliot et al. [15] was first translated from English to Kannada (the local language) and then back-translated to English by another person who was fluent in both languages to check for correct translation. The questionnaire was well understood by the participants. However, the pilot study findings showed that seven out of ten participants were not using a helmet and used a mobile phone while driving, which confirms earlier research that suggests that motorcyclists use mobile phones while driving [26]. Also, inconsistent helmet usage is prevalent, especially in the coastal cities of Western India, because of the humid weather conditions [27, 28]. Therefore, it was decided to add questions about helmet use and mobile phone usage to the modified version of the MRBQ. It should be noted that the original MRBQ does not have any questions on helmet and mobile phone usage.

Besides the MRBQ items, the questionnaire assessed motorcyclist’s socio-demographics, motorcycle ownership details, crash involvement, and fines paid. The first component included information on socio-demographic variables such as age, gender, education, and occupation. For the details regarding their motorcycles and driving, participants provided information on the type of motorcycle and the average riding hours in a week. Participants were also asked to provide information on their crash history during the past three months, including severe crash injuries (1 = none, 2 = one, 3 = two or more), mild crash injury (1 = none, 2 = one, 3 = two or more), and near-crash experience (1 = none, 2 = one, 3 = two or more).

### Data analysis

The data were entered and subsequently analysed using IBM’s statistical package SPSS version 22. The factor structure of the MRBQ was determined using exploratory factor analysis with Principal Axis Factoring (PAF) and direct Oblimin method. The associations between MRBQ factors and crash-related outcomes were explored using univariate and multivariate binary logistic regression. The univariate analysis is relevant for determining possible targets for future intervention programs, while the multivariate analysis provides information about the unique contribution of each factor in predicting crash experience and receiving fines while controlling for the other MRBQ factors and the overall variance explained [29]. To this end, the outcome variables were recoded into a binary variable with no = 0, if no crash or near-crash involvement is reported or no fines have been paid, and yes =1, if participants reported one or more crash or near-crashes and fines paid. The cut off for *p*-value was set at 0.05, and the odds ratio for each independent variable was calculated at a 95% confidence interval.

## Results

### Participant characteristics

Out of the 300 participants, 52.7% were in the age group of 18–20 years, followed by 32 and 15.3% in the age range of 21–23 and 24–25 years respectively (M = 20.91; SD = 2.06) (see Table 1). The majority of the respondents (93%) in the study were males, which is in line with observations that suggest there are fewer female than male riders overall, particularly on higher-powered non-moped/scooter type models [3]. Females usually ride a moped or a gearless scooter. Almost all (92.3%) participants were college students. Out of the 300 participants, 57.6% rode motorcycles with a power range between 125 cc to 200 cc, and 40.7% rode motorcycles between 100 cc to 125 cc. Just 1.7% % of the participants reported riding motorcycles with more than 500 cc. Weekly, 46.3% of the participants, rode less than 5 h, and 36.4 and 17.3% of them reported average hours of riding between 5 and 10 h and more than 10 h, respectively.

**Table 1:** Participant characteristics & crash experience

Age	% (n)	Mean	Standard deviation
18–20	52.7 (159)	20.91	2.06
21–23	32 (96)		
24–25	15.3 (45)		
**Gender**	**%(n)**		
Male	93 (279)		
Female	7 (21)		
**Education**	**%(n)**		
Illiterate	1.3 (4)		
SSLC	3.7 (11)		
Graduate	82.3 (247)		
Postgraduate	12.7 (38)		
**Category**	**%(n)**		
Student	92.3 (277)		
Employed	7 (21)		
Unemployed	0.7 (2)		
**Type of Motorcycle**	**%(n)**		
100 cc–125 cc	40.7(122)		
125 cc–200 cc	57.6 (173)		
More than 500 cc	1.7 (5)		
**Average hours of riding in a week**			
**Number**	**%(n)**		
Less than 5 h	46.3 (139)		
5–10 h	36.4 (109)		
> 10 h	17.3 (52)		
**The number of severe injury crashes they were involved in over the past three months**			
**Number**	**%(n)**		
None	74.7 (224)		
One	17 (51)		
vTwo or more	8.3 (25)		
**Light/mild injury crashes**			
**Number**	**%(n)**		
None	57.7 (173)		
One	36.7 (110)		
Two or more	5.7 (17)		
**Material damage crash (Damage to some part of the motorcycle)**			
**Number**	**%(n)**		
None	53.7 (161)		
One	35 (105)		
Two or more	11.3 (34)		
**Near crash experience over the past three months**			
**Number**	**%(n)**		
None	52.7 (158)		
One	34.3 (103)		
Two or more	13 (39)		

### Crash experience

Table 1 also describes the crash experiences of study participants. The majority of the participants (74.7%) were not involved in any severe injury crashes over the past three months. However, 42.4% of the participants got one or more mild injuries, and 46.3% experienced damage to some part of their motorcycle during the past three months. Out of the total participants, 47.3% experienced near-crash experience once or more over the past three months.

### Factor analysis of the MRBQ

The factor analysis was performed to identify the optimal factor structure of the MRBQ in the Indian setting (see Table 2). The exploratory analysis of the MRBQ items revealed five factors (loading cut-off point = 0.3). The original five-factor names were retained for the present factors due to their similarity with previously reported factors [15]. Seven items loaded on factor 1‘traffic errors’ (α = .66), nine items loaded on factor 2 ‘control errors’ (α = .69), nine items loaded on factor 3 ‘usage of protective equipment’ (α = .82), two items loaded on factor 4 ‘stunts’ (α = .79), and, finally nine items loaded on factor 5 ‘violations’ (α = .80). The items with low factor loadings (<.30) were omitted from the model. Among the five identified factors, protective equipment, violations, and stunts have a good internal consistency of .82, .80, and .79, respectively. The total Cronbach’s alpha of the MRBQ scale was .80, which indicates a good internal consistency and suggests that all the factors measure a common underlying theme (i.e., risky riding behaviour). The factor traffic errors explained the largest share of variance in the data (15%), while the other factors control errors, protective equipment, stunts, and violations accounted for 10, 6, 5, and 3% of the variance, respectively (Table 2).

**Table 2:** Factor structure and loadings of the MRBQ items

Questions	1	2	3	4	5
	Traffic errors	Control errors	Protective equipment	Stunts	Violations
1. Drive the vehicle so fast into a corner (or curve) until you feel that you might lose control	0.30				
2. When riding at the same speed as other traffic, when the traffic light indicates to stop, it becomes difficult to stop in time	0.44				
3. Going quite wide from the corner of the road when negotiating a corner	0.47				
4. Failing to notice a pedestrian waiting at a crossing when the lights have just turned red	0.52				
5. Failing to notice the pedestrians are crossing when turning onto a side street from the main road	0.43				
6. Attempting to overtake someone without noticing a right turn signal	0.38				
7. Driving in between two lanes of fast-moving traffic	0.37				
8. Go onto the main road in front of a vehicle that you did not notice or whose speed you had misjudged		0.30			
9. Did not notice or anticipate another vehicle coming in front of you and had difficulty to stop		0.39			
10. Being distracted or pre-occupied, you suddenly realize that the vehicle in front has slowed, and you have to apply the brake hard to avoid a collision		0.37			
11. Not noticing someone stepping out from a parked vehicle and it is too late for you to stop your vehicle to avoid collision with him		0.49			
12. Go extremely fast towards a corner that you will feel scared		0.34			
13. While waiting for your turn to turn left on the main road, you pay such close attention to the main traffic that you are almost about to hit a vehicle that is in your front		0.43			
14. Need to brake or back-off when negotiating a bend		0.39			
15. Skidding on wet road or manhole cover, road marking, etc.		0.52			
16. You go so close to the vehicle at the front that it becomes difficult to stop in an emergency		0.53			
17. Use motorcycle protective trousers (leather or non-leather)			0.69		
18. Use motorcycle boots			0.77		
19. Use motorcycle protective jacket (leather or non-leather)			0.83		
20. Use body armor/shock protectors (e.g., elbow, shoulder, knee)			0.78		
21. Bright/fluorescent stripes/patches on your clothing			0.72		
22. Use Leather one-piece motorcycle suit			0.57		
23. Use Bright/fluorescent clothing			0.45		
24. Use motorcycle gloves			0.74		
25. Do you wear any motorcycle-specific protective clothing			0.69		
26. Attempt or done a wheelie				−0.37	
27. Intentionally doing a wheel spin				−0.42	
28. Exceed the speed limit in a motorway					--0.59
29. Exceed the speed limit on rural roads					−0.53
30. Exceed the speed limit in a residential road (Colony road)					0.41
31. Talk on the mobile phone while riding					0.75
32. Texting and driving					0.66
33. Ignore the speed limit at late night or early morning					−0.61
34. Going very fast in a country road					−0.67
35. Engage in racing with other riders or drivers					−0.45
36. Going fast from the traffic lights to defeat the driver/rider in front of you					−0.36
**α**	0.66	0.69	0.82	0.79	0.80
*R*^*2*^	0.15	0.10	0.6	0.5	0.3
*Mean*	2.02	2.26	5.07	1.49	2.24
***SD***	0.67	0.62	1.01	0.90	0.82

There were some differences among the factor loadings when comparing the original MRBQ context [15] to the modified MRBQ in the Indian context (see Table S1). Two items (“driving in between two lanes of fast-moving traffic”; “go extremely fast towards a corner that you will feel scared”), which loaded onto the stunts factor for the UK participants, but loaded under traffic errors and control errors respectively among the Indian participants. Two items that were added to the original MRBQ to assess mobile phone usage (“talk on the mobile phone while riding,” “texting and driving”) while driving loaded onto the violations factor in the current study. Three items related to helmet usage (“use helmet while riding,” “comfortable in using helmet” & “not using helmet while riding slowly”) that were added to the original MRBQ were omitted from the model due to low factor loadings (<.30). Also, one item (engage in racing with other riders or drivers) loaded under violations in the current study, but loaded under stunts in the original study conducted by Elliot et al. in 2007 [15].

Table S2 shows the correlations among the MRBQ factors. Traffic errors showed strong positive correlations with control errors, stunts, and violations, whereas it was weaker and negatively correlated with protective equipment. Strong positive correlations were further found for violations with control errors and stunts. Protective equipment showed moderate negative associations with control errors and stunts.

### Factors associated with crash involvement in the last three months

Table 3 shows the results of the regression analysis for crash involvement in the last three months. There was no association between crash involvement and the type of motorcycle, (*Wald* (2, *N* = 300) = 2.81, *p* = .25), and average hours of riding in a week (*Wald* (2, *N* = 300) =1.09, *p* = .58). The MRBQ stunt factor was dummy coded into less or equal to median and more than median. The factors traffic errors, control errors, violations, and protective equipment were categorized into lower, second, third, and upper quartile. No significant associations were found between the MRBQ factors and crash involvement in the last three months (stunts: (*Wald* (1, *N* = 300) = 3.30, *p* = .07); (traffic errors: (*Wald* (3, N = 300) = 2.81, *p* = .42); (control errors: (*Wald* (3, N = 300) = 3.69, *p* = .30); (violations: (*Wald* (3, N = 300) = 4.84, *p* = .18). There was a significant negative association between wearing protective equipment and severe crash involvement (*Wald* (3, N = 300) = 8.22, *p* = .04). Posthoc comparisons showed that participants who scored more in the second and third quartile were less likely to be involved in severe crashes. Multiple logistic regression was not performed in this case because only one variable (protective equipment) was statistically significant in the univariate analysis.

**Table 3:** Factors associated with severe crash injury over the past three months

Variable	Crashed %	Not crashed %	OR	95% CI	***p***-value
**Type of motorcycle**					
100 cc–125 cc	23.8	76.2	Referent		
125 cc–200 cc	25.4	74.6	0.91	(0.53–1.57)	0.74
More than 500 cc	60	40	0.21	(0.03–1.31)	0.09
**Average hours of riding in a week**					
< 5 h	26.6	73.4	Referent		
5–10 h	22	78	1.29	(0.71–2.32)	0.40
> 10 h	15	37	0.89	(0.44–1.82)	0.76
**Stunts (MRBQ)**					
**Stunts**	**Yes**	**No**	**OR**	**95% CI**	***p*-value**
Less than or equal to median	46.8	53.2	Referent		
More than median	55.7	44.3	1.64	(0.96–2.83)	0.07
**Traffic errors (MRBQ)**					
**Traffic errors**	**Yes**	**No**	**OR**	**95% CI**	***p*-value**
Lower quartile	46.7%	53.3%	Referent		
Second quartile	47.1%	52.9%	0.63	(0.31–1.27)	0.19
Third quartile	55.2%	44.8%	1.09	(0.53–2.26)	0.81
Upper quartile	51.8%	48.2%	0.94	(0.43–2.05)	0.88
**Control errors (MRBQ)**					
**Control errors**	**Yes**	**No**	**OR**	**95% CI**	***p*-value**
Lower quartile	37.8%	62.2%	Referent		
Second quartile	53.3%	46.7%	1.42	(0.69–2.92)	0.35
Third quartile	58.1%	41.9%	1.32	(0.59–2.92)	0.5
Upper quartile	51.5%	48.5%	2.06	(0.98–4.36)	0.06
**Violations (MRBQ)**					
**Violations**	**Yes**	**No**	**OR**	**95% CI**	***p*-value**
Lower quartile	44.3%	55.7%	Referent		
Second quartile	42.9%	57.1%	0.86	(0.36–2.06)	0.73
Third quartile	51.9%	48.1%	1.75	(0.88–3.48)	0.11
Upper quartile	59.0%	41.0%	1.65	(0.76–3.58)	0.21
**Protective equipment (MRBQ)**					
**Protective equipment**	**Yes**	**No**	**OR**	**95% CI**	***p*-value**
Lower quartile	36.7%	63.3%	Referent		
Second quartile	19.0%	81.0%	0.41	(0.19–0.88)	0.02*
Third quartile	19.6%	80.4%	0.42	(0.21–0.83)	0.01*
Upper quartile	26.2%	73.8%	0.61	(0.29–1.27)	0.19

Note: *significant at *p* < .05

### Factors associated with near-crash experience over the past three months

Table 4 shows the results of the regression analysis for near-crash experience over the past three months. There was no association between near-crash experience and the type of motorcycles (*Wald* (2, *N* = 300) = 1.45, *p* = .48), No significant associations were found for traffic errors (*Wald* (3, *N* = 300) = 3.18, *p* = .37) and wearing protective equipment (*Wald* (3, N = 300) = 4.16, *p* = .25) with near-crash experiences. However, stunts, control errors, violations and average hours of riding in a week (5–10 h) showed a significant association with near-crash experiences (stunts: *Wald* (1, N = 300) = 8.79, *p* = .01) (control errors: (*Wald* (3, N = 300) = 6.67, *p* = .04) (violations: *Wald* (3, N = 300) = 12.49, *p* = .01) (Average hours of riding in a week: *Wald* (2, *N* = 300) =6.22, *p* = .04). Posthoc comparisons showed that participants scoring in the third and upper quartile on violations were more likely to be involved in near-crash experiences over the past three months as well as those performing more stunts. Multiple logistic regression included those sociodemographic variables and MRBQ factors that showed significant univariate associations with near-crash involvement, namely average hours of riding, stunts, control error, violation and protective equipment. Among these, stunts (*Wald* (1, *N* = 300) = 7.42, *p* = 0.04) and violations (*Wald* (3, N = 300) = 10.54, *p* = 0.04) showed unique significant associations with near-crash involvement (see Table 4).

**Table 4:** Factors associated with near-crash experience over the past three months

**Variable**	**Crashed %**	**Not crashed %**	**OR (crude)**	**95% CI (crude)**	***p*-value (crude)**	**OR (adj)**	**95% CI (adj)**	***p*-value (adj)**
**Type of motorcycle**								
100 cc–125 cc	23.8%	76.2%	Referent					
125 cc–200 cc	25.4%	74.6%	1.15	(0.19–7.14)	0.88	–	–	–
More than 500 cc	60%	40%	1.51	(0.25–9.31)	0.65	–	–	–
**Average hours of riding in a week**								
< 5 h	26.6%	73.4%	Referent					
5–10 hours	22%	78%	**1.81**	(1.08–2.98)	0.02*	1.65	(0.95–2.85)	0.07
> 10 h	28.8%	71.2%	1.78	(0.94–3.39)	0.07	1.52	(0.76–3.01)	0.24
**Stunts (MRBQ)**								
**Stunts**	**Yes**	**No**	**OR**	**95% CI**	***p*-value**	**OR (adj)**	**95% CI (adj)**	***p*-value (adj)**
Less than or equal to median	41.4%	58.6%	Referent					
More than median	59.8%xz	40.2%	**2.11**	(1.29–3.45)	0.01*	**1.74**	**(1.03–2.44)**	0.04*
**Traffic errors (MRBQ)**								
**Traffic errors**	**Yes**	**No**	**OR**	**95% CI**	***p*-value**	**OR (adj)**	**95% CI (adj)**	***p*-value (adj)**
Lower quartile	48%	52%	Referent					
Second quartile	46.1%	53.9%	0.93	(0.51–1.68)	0.8	–	–	–
–	55.2%	44.8%	1.34	(0.69–2.59)	0.39	–	–	–
Upper quartile	39.3%	60.7%	0.7	(0.35–1.41))	0.32	–	–	–
**Control errors (MRBQ)**								
**Control errors**	**Yes**	**No**	**OR**	**95% CI**	***p*-value**	**OR (adj)**	**95% CI (adj)**	***p*-value (adj)**
Lower quartile	39%	61%	Referent					
Second quartile	53.3%	46.7%	1.78	(0.97–3.28)	0.06	0.76	(0.39–1.47)	0.41)
Third quartile	56.5%	43.5%	**2.02**	(1.04–3.96)	0.04*	0.66	(0.32–1.37)	0.27
Upper quartile	40.9%	59.1%	1.08	(0.56–2.10)	0.82	1.75	(0.81–3.75)	0.15
**Violations (MRBQ)**								
**Violations**	**Yes**	**No**	**OR**	**95% CI**	***p*-value**	**OR (adj)**	**95% CI (adj)**	***p*-value (adj)**
Lower quartile	36.7%	63.3%	Referent					
Second quartile	35.7%	64.3%	0.96	(0.47–1.95)	0.91	1.21	(0.57–2.56)	0.63
Third quartile	53.8%	46.2%	**2.01**	(1.11–3.66)	0.02*	0.57	(0.3–1.07)	0.08
Upper quartile	60.7%	39.3%	**2.66**	(1.34–5.29)	0.01*	**2.08**	**(1.08–4.68)**	0.03*
**Protective equipment (MRBQ)**								
**Protective equipment**	**Yes**	**No**	**OR**	**95% CI**	***p*-value**	**OR (adj)**	**95% CI (adj)**	***p*-value (adj)**
Lower quartile	44.3%	55.7%	Referent					
Second quartile	52.4%	47.6%	0.72	(0.37–1.40)	0.34	0.78	(0.38–1.62)	0.51
Third quartile	59.8%	40.2%	**0.53**	(0.29–0.98)	0.04*	0.55	(0.29–1.06)	0.07
Upper quartile	52.5%	47.5%	0.72	(0.37–1.41)	0.72	0.85	(0.4–1.81)	0.68

Note: *significant at *p* < .05

### Fines paid due to traffic violations in the last three months

Table 5 shows the results of the regression analysis for fines paid due to a traffic violation in the last three months. There was no association between fines paid and average hours of riding in a week (*Wald* (2, *N* = 300) = 2.56, *p* = .28). There was a significant association between type of motorcycles (125 cc–200 cc) and fines paid (*Wald* (2, *N* = 300) = 5.81, *p* = .01). Posthoc comparisons showed that those riding a motorcycle of 125 cc–200 cc were more likely to have paid a fine in the last three months than those with lighter motorcycles. This difference was not found among those with heavier motorcycles. No significant associations were found between wearing protective equipment and fines paid (protective equipment: *Wald* (3, *N* = 300) = 2.61, *p* = .46). However, there were significant associations between MRBQ factors of stunts, traffic errors, control errors and violations and fines paid (stunts: *Wald* (1, *N* = 300) = 3.93, *p* = .04); (traffic errors: *Wald* (3, *N* = 300) = 9.94, *p* = .02; (control errors: *Wald* (3, *N* = 300) = 9.29, *p* = .02); (violations: *Wald* (3, N = 300) = 8.86, *p* = .03). Posthoc comparisons showed that participants scoring in the upper quartile for traffic errors, second and upper quartile for control errors, third and upper quartile for violations, and those performing more stunts were more likely to pay fines due to traffic violations. Multiple logistic regression was performed with the variables which were significant in the univariate analysis, namely type of motorcycles, stunts, traffic error, control error, and violations. Type of motorcycles (*Wald* (2, N = 300) = 4.81, *p* = .02), stunts (*Wald* (1, N = 300) = 3.527, *p* = .04), and violations (*Wald* (3, N = 300) = 8.802, *p* = .04) showed unique significant associations with fines paid (see Table 5).

**Table 5:** Factors associated with fines paid for traffic violation in the last three months

Variable	Paid %	Not Paid %	OR	95% CI	***p***-value	OR (adj)	95% CI (adj)	***p***-value (adj)
**Type of motorcycle**								
100 cc–125 cc	28.7%	71.3%	Referent					
125 cc–200 cc	41.0%	59.0%	**1.73**	1.05–2.84	0.03*	**1.53**	**(1.10–2.59)**	0.01*
More than 500 cc	60.0%	40.0%	0.27	0.04–1.67	0.16	2.17	(0.32–14.92)	0.43
**Average hours of riding in a week**								
< 5 h	31.7%	68.3%	Referent					
5–10 h	39.4%	60.6%	1.59	0.82–3.05	0.17	–	–	–
> 10 h	42.3%	57.7%	1.12	0.56–2.20	0.73	–	–	–
**Stunts (MRBQ)**								
**Stunts**	**Yes**	**No**	**OR**	**95% CI**	***p*-value**	**OR (adj)**	**95% CI (adj)**	***p*-value (adj)**
Less than or equal to median	22.2%	77.8%	Referent					
More than median	32%	68%	**1.65**	(1.01–2.72)	0.04*	**1.16**	**(1.02–2.04)**	0.03*
**Traffic errors (MRBQ)**								
**Traffic errors**	**Yes**	**No**	**OR**	**95% CI**	***p*-value**	**OR (adj)**	**95% CI (adj)**	***p*-value (adj)**
Lower quartile	28%	72%	Referent					
Second quartile	19.6%	80.4%	0.79	(0.42–1.51)	0.48	1.58	(0.80–3.13)	0.19
Third quartile	29.9%	70.1%	1.19	(0.6–2.37)	0.62	1.24	(0.57–2.69)	0.58
Upper quartile	26.8%	73.2%	**2.31**	(1.13–4.70))	0.02*	1.24	(0.51–3.00)	0.61
**Control errors (MRBQ)**								
**Control errors**	**Yes**	**No**	**OR**	**95% CI**	***p*-value**	**OR (adj)**	**95% CI (adj)**	***p*-value (adj)**
Lower quartile	19.5%	80.5%	Referent	.				
Second quartile	25.6%	74.4%	**1.97**	(1.02–3.81)	0.04*	1.72	(0.84–3.50)	0.14
Third quartile	24.2%	75.8%	1.7	(0.83–3.52)	0.15	1.37	(0.62–3.01)	0.44
Upper quartile	33.3%	66.7%	**2.92**	(1.45–5.86)	0.01*	1.96	(0.83–4.65)	0.13
**Violations (MRBQ)**								
**Violations**	**Yes**	**No**	**OR**	**95% CI**	***p*-value**	**OR (adj)**	**95% CI (adj)**	***p*-value (adj)**
Lower quartile	25.3%	74.7%	Referent					
Second quartile	30.4%	69.7%	1.29	(0.6–2.76)	0.51	1.10	(0.49–2.44)	0.81
Third quartile	42.3%	57%	**2.16**	(1.14–4.10)	0.02*	1.71	(0.85–3.41)	0.13
Upper quartile	45.9%	54.1%	**2.5**	(1.22–5.11)	0.01*	**1.47**	**(1.26–3.36)**	0.03*
**Protective equipment (MRBQ)**								
**Protective equipment**	**Yes**	**No**	**OR**	**95% CI**	***p*-value**	**OR (adj)**	**95% CI (adj)**	***p*-value (adj)**
Lower quartile	36.7%	63.3%	Referent					
Second quartile	19%	81%	0.62	(0.31–1.23)	0.17	–	–	–
Third quartile	19.6%	80.4%	0.65	(0.35–1.2)	0.17	–	–	–
Upper quartile	26.2%	73.8%	0.8	(0.4–1.59)	0.52	–	–	–

Note: *significant at p < .05

## Discussion

The present study aimed to determine the most appropriate factor structure of the modified version of the MRBQ among the motorcyclists in Manipal, India, and secondly, to assess which MRBQ factors are associated with self-reported crash involvement and violations.

### The MRBQ factor structure

The exploratory factor analysis for the MRBQ questionnaire revealed a 36-item five-factor solution, namely traffic errors, control errors, stunts, protective equipment, and violations. The five-factor structure that emerged out of the current study was similar to the factor structure in studies conducted in Australia, the UK, and Turkey, respectively [14, 15, 23]. Out of the five identified factors, protective equipment, violations, and stunts, reported a good internal consistency. Traffic errors contained seven items, control errors and protective equipment nine items each, stunts contained two items, and violations had nine items. The items added to assess mobile phone usage behaviour while riding got loaded under the violations factor. A significant proportion of the participants talk (25.3%) and text (34.8%) on their mobile phones while riding. Globally, these findings are consistent with the studies conducted in Mexico and Vietnam. The study results in Mexico showed that the prevalence of mobile phone usage was high among motorcyclists of all age groups [30]. Similar results were also reported in Vietnam, where high mobile phone usage was reported among young motorcyclists [31]. From the Indian perspective, these results align with studies conducted by Save LIFE Vodafone Foundation and in Kerala, India that reported high usage of mobile phones among young riders while riding [26, 28]. The three items related to helmet usage (i.e., use helmet while riding, comfortable in using helmet, and not using helmet while riding slowly) added in first instance to the original MRBQ were omitted from the model due to low factor loadings. Wearing a helmet is mandatory in India for two-wheelers. However, studies conducted by Thajudeen et al. and Sreedharan et al. reported low helmet usage in settings similar to the current study setting [27, 28]. This is in line with a study conducted among Iranian motorcyclists where it was reported that 67% of the motorcyclists do not use helmet while riding [32]. In a study conducted by Fletcher et al. in 2019 in Jamaica, low helmet usage (29.4%) was reported among young motorcyclists [33]. This is consistent with the findings of a study conducted in Ghana, where 34.2% of the respondents use a helmet while riding [34]. Furthermore, in our pilot study seven out of ten respondents reported not to use a helmet while riding or only very inconsistently, which was the reason for adding the three items on helmet usage (“use helmet while riding,” “comfortable in using helmet” & “not using helmet while riding slowly”) to the original MRBQ. However, in our main study, 89% of the respondents reported that they use a helmet while riding at all times and also feel comfortable using the helmet. Maybe because of reasons related to socially desirable answering, but respondents were very positive about helmet usage. As a result, the variation in this variable was low, which may have resulted in the low contribution of the items to the explanation of the factor protective equipment and their non-significance in the predictions of crash experience.

Overall, the item loadings for the traffic errors factor were consistent with MRBQ studies conducted in Australia, the UK and Nigeria [14, 15, 25], except for the item “driving in between two lanes of fast-moving traffic”, which originally loaded onto the stunts factor in the study conducted by Elliott et al. [15] but in the current study, loaded under traffic errors. Riding in between two lanes of fast-moving traffic is considered dangerous on Indian roads and the offender can be booked under the reckless driving act [35]. It could therefore be argued that the young respondents rated this item similar to other errors due to lack of experience [36].

The two items loading onto the stunts factor in the present study confirmed the findings from the previous study conducted by Elliott et al. [15]. However, the items “driving in between two lanes of fast-moving traffic,” “go extremely fast towards a corner that you feel scared” and “engage in racing with other riders or drivers”, which were loaded onto stunts factor in the study conducted by Elliott et al. [15], were loaded under traffic errors, control errors and violations, respectively, in the present study. Similar to the study conducted by Save life foundation [26] on distracted riding this can be attributed to the fact that the study respondents would have rated these items similar to other errors items due to lack of awareness regarding its danger or distracted riding which warrants further exploration.

For the control error factor, it was evident that item loadings onto this factor covered behaviours that were likely to be non-intentional (e.g., “skid on a wet manhole cover”) and related to speeding, careless riding, and inattentiveness (e.g., “You go so close to the vehicle at the front that it becomes difficult to stop in an emergency”). The findings of the present study confirmed the findings from the previous study conducted by Elliott et al. [15].

Lastly, in the current study, a factor related to protective equipment emerged from the data. Most of the participants have rarely used any form of protective equipment during motorcycle riding. This suggests that in India, motorcycle riders hardly use protective equipment during their day-to-day commutation.

### Correlates of crash experiences

Performing stunts was positively associated with self-reported near-crash experiences over the past three months. Riders who reported attempting or have done a wheelie and intentionally spinning the wheel had more chance of getting involved in near-crash experiences. These findings were similar to findings reported by the studies conducted [14, 23] in Australia and Turkey. The results also align with previous studies that identified stunts behaviour as the cause of crash involvement in both police reports [24] and self-reported incidences of crashes [14]. Beside performing stunts, riders who reported violations also had more risk of getting involved in recent near-crash experiences, which aligns with previous studies highlighting the positive association between violations and recent near-crash experiences [14, 15, 23].

In the current study, type of motorcycle, stunts and violations were positively associated with fines paid because of committing traffic violations in the last three months. Riders using motorcycles (125-200 cc), performing stunts and reporting frequent violations are more likely to pay fines compared to the riders who reported using of low-powered motorcycles, not performing stunts and committing traffic violations. These results align with previous studies’ findings [14, 15] conducted in Australia and the United Kingdom, where it was reported that performing stunts and committing traffic violations was associated with paying fines. In the Southeast Asian context, this finding is similar to a study conducted in Indonesia, which reported that young aged motorcyclists are more prone to violating traffic rules and paying fines [37]. In a study conducted by Dandona et al. [38] in Hyderabad, India, it was found that more than one-fourth of the respondents have been penalized by traffic police for violating traffic rules. There was no association between stunts, traffic error, control error and violations with severe crash injury in the last three months. This resembles the findings of a study by Stephens et al. [14] in Victoria, Australia, where no significant association was found between traffic error, control error and violations with severe crash injury. However, the study by Stephens et al. reported that stunts behaviour had a significant association with severe crash injury. In the present study among all the MRBQ factors, only wearing protective equipment was (negatively) associated with severe crash involvement. This corresponds to findings reported by De Rome et al. in a study conducted in the Australian Capital Territory [39] and Erdogan et al. in Turkey [40]. In both the studies, it was reported that using protective equipment significantly reduces the risk of injury in crash involvement. For the present study, performing stunts was not directly associated with severe crash involvement; given its association with near-crashes, there is likely to be an indirect association between stunts behaviour and crash risk. This finding is suggestive of further investigation.

### Practical implications

As mentioned in the above, the current modified version of MRBQ can be a suitable version of MRBQ that can be used in other settings of India. The modified MRBQ can be an effective tool to investigate risky riding behaviour among at-risk young motorcyclists in India to target for intervention. The present study shows that performing stunts and reporting traffic violations were the two MRBQ factors positively associated with recent near-crash experiences among young motorcyclists. In case these findings are replicated in future research, it is thus recommended for the local policymakers to initiate targeted interventions that focus on the predictors of risky driving to reduce crash and injury rates. The current study’s findings have also generated evidence for the local authorities about the importance of strict law enforcement for traffic violations. In addition, the findings of the current study may be of value for decision-makers to implement strict regulations for motorcyclists riding underage or without a proper valid license because unless the licensing procedure in India is regulated and closely monitored, the quality of the rider will be questionable [22, 41].

### Limitations and future research

The focus of the study was very specific both in terms of place (i.e., Manipal) and age (i.e., young riders). Therefore, the results of this study do not simply generalize to other places and age groups. The present study needs further replication using a larger population and broader age group involvement to come to a more generalizable overview of the factor structure of the MRBQ in the Indian context. Nevertheless, internationally, the young rider population is a very important focus in traffic safety research. The other main limitation of the study was the fact that we made use of self-reported data, which could have promoted socially desirable responses [24, 42]. The key findings that emerged out of the study were that stunts and violations were the two MRBQ factors positively associated with recent near-crash experiences, which require further exploration in explicitly investigating these factors for near-crash involvement in a broader population, and why no associations were found with severe crash involvement although that may have been caused by the lower number of participants that experienced a severe crash.

## Conclusion

In this study, the factor structure of a modified version MRBQ and the extracted factor’s associations with self-reported crash involvement were assessed. Five factors, namely traffic errors, control errors, violations, stunts, and protective equipment, emerged from the modified MRBQ scale. The factor structure revealed in the current study is consistent with MRBQ factor structures found in other countries [14, 15, 23]. To the best of our knowledge, the MRBQ has not been tested in India yet. The current study contributes to the existing literature and knowledge regarding the understanding of risky motorcycle rider behaviour among young motorcyclists in India. However, the use of a convenience sample warrants further studies using the modified version of MRBQ in other settings of India to support more general use of the MRBQ in the wider Indian context to understand and evaluate motorcyclists ‘risky behaviour. If replicated by future studies, local policymakers are advised to focus on these findings while identifying risky riding behaviours and the subsequent planning of behavioural, infrastructural and policy interventions to achieve a reduction in the number of road crashes among motorcyclists.

## Supplementary Information

**Additional file 1.**

## Data Availability

The datasets generated during this study are not available for the sensitive and personal nature of the information contained. Data may be available upon justified request from the corresponding author with restrictions and following the ethical approval.

## Abbreviations

DBQ: Driver behaviour questionnaire
LMIC: Low and middle-income countries
MRBQ: The motorcycle rider behaviour questionnaire
PAF: Principal Axis Factoring
