# Supplementary material for: Risky motorcycle riding behaviour among young riders in Manipal, India
Source: BMC Public Health. 2021 Oct 28;21:1954. doi: 10.1186/s12889-021-11899-y (PMC8555150; doi:10.1186/s12889-021-11899-y)
Supplement: Supplementary file 1 — Additional file 1. [file 12889_2021_11899_MOESM1_ESM.docx]

Table S1: Factor structure of the original MRBQ (Elliot et al., 2007) compared to the present study in the Indian context

| **Questions** | **Original**  **MRBQ** | **Modified MRBQ** |
| --- | --- | --- |
| 1. Drive the vehicle so fast into a corner (or curve) until you feel that you might lose control | Traffic errors | Traffic errors |
| 1. When riding at the same speed as other traffic, when the traffic light indicates to stop, it becomes difficult to stop in time | Traffic errors | Traffic errors |
| 1. Going quite wide from the corner of the road when negotiating a corner | Traffic errors | Traffic errors |
| 1. Failing to notice a pedestrian waiting at a crossing when the lights have just turned red | Traffic errors | Traffic errors |
| 1. Failing to notice the pedestrians are crossing when turning onto a side street from the main road | Traffic errors | Traffic errors |
| 1. Attempting to overtake someone without noticing a right turn signal | Traffic errors | Traffic errors |
| 1. Driving in between two lanes of fast-moving traffic | Stunts | Traffic errors |
| 1. Go onto the main road in front of a vehicle that you did not notice or whose speed you had misjudged | Control errors | Control errors |
| 1. Did not notice or anticipate another vehicle coming in front of you and had difficulty to stop | Control errors | Control errors |
| 1. Being distracted or pre-occupied, you suddenly realize that the vehicle in front has slowed, and you have to apply the brake hard to avoid a collision | Control errors | Control errors |
| 1. Not noticing someone stepping out from a parked vehicle and it is too late for you to stop your vehicle to avoid collision with him | Control errors | Control errors |
| 1. Go extremely fast towards a corner that you will feel scared | Stunts | Control errors |
| 1. While waiting for your turn to turn left on the main road, you pay such close attention to the main traffic that you are almost about to hit a vehicle that is in your front | Control errors | Control errors |
| 1. Need to brake or back-off when negotiating a bend | Control errors | Control errors |
| 1. Skidding on wet road or manhole cover, road marking, etc. | Control errors | Control errors |
| 1. You go so close to the vehicle at the front that it becomes difficult to stop in an emergency | Control errors | Control errors |
| 1. Use motorcycle protective trousers (leather or non-leather) | Protective equipment | Protective equipment |
| 1. Use motorcycle boots | Protective equipment | Protective equipment |
| 1. Use motorcycle protective jacket (leather or non-leather) | Protective equipment | Protective equipment |
| 1. Use body armor/shock protectors (e.g., elbow, shoulder, knee) | Protective equipment | Protective equipment |
| 1. Bright/fluorescent stripes/patches on your clothing | Protective equipment | Protective equipment |
| 1. Use Leather one-piece motorcycle suit | Protective equipment | Protective equipment |
| 1. Use Bright/fluorescent clothing | Protective equipment | Protective equipment |
| 1. Use motorcycle gloves | Protective equipment | Protective equipment |
| 1. Do you wear any motorcycle-specific protective clothing | Protective equipment | Protective equipment |
| 1. Attempt or done a wheelie | Stunts | Stunts |
| 1. Intentionally doing a wheel spin | Stunts | Stunts |
| 1. Exceed the speed limit in a motorway | Violations | Violations |
| 1. Exceed the speed limit on rural roads | Violations | Violations |
| 1. Exceed the speed limit in a residential road (Colony road) | Violations | Violations |
| 1. Talk on the mobile phone while riding | New addition | Violations |
| 1. Texting and driving | New addition | Violations |
| 1. Ignore the speed limit at late night or early morning | Violations | Violations |
| 1. Going very fast in a country road | Violations | Violations |
| 1. Engage in racing with other riders or drivers | Stunts | Violations |
| 1. Going fast from the traffic lights to defeat the driver/rider in front of you | Violations | Violations |

Table S2: Correlations between MRBQ factors

|  | **Traffic errors** | **Control errors** | **Protective Equipment** | **Stunts** | **Violations** |
| --- | --- | --- | --- | --- | --- |
| Traffic errors |  | 0.65* | -0.25* | 0.32* | 0.45* |
| Control errors |  |  | -0.27* | 0.23* | 0.39* |
| Protective Equipment |  |  |  | -0.19* | -0.06 |
| Stunts |  |  |  |  | 0.46* |
| Violations |  |  |  |  |  |

Note: *p < 0.05
